# Video-based teach-to-goal intervention on inhaler technique on adults with asthma and COPD: A randomized controlled trial

**DOI:** 10.1371/journal.pone.0286870

**Published:** 2023-06-09

**Authors:** Mohammad Samer Al-Kharouf, Mariam Hantash Abdeljalil, Nathir M. Obeidat, Khaled Al Oweidat, Oriana Awwad

**Affiliations:** 1 Department of Biopharmaceutics and Clinical Pharmacy, School of Pharmacy, The University of Jordan, Amman, Jordan; 2 Faculty of Medicine, Department of Internal Medicine, The University of Jordan, Amman, Jordan; 3 Department of Respiratory and Sleep Medicine, Jordan University Hospital, Amman, Jordan; Jordan University of Science and Technology, JORDAN

## Abstract

**Background:**

Incorrect use of inhalers is a problem associated with poor patient outcomes. Despite improvement in the technique after verbal educations, this deteriorates over-time requiring re-enforcement through different educative strategies. This study aimed to assess the impact of a novel video-based teach-to-goal (TTG) educational intervention on: mastery of inhaler technique, disease control, medication adherence and disease-related quality of life (QoL) over-time among asthma and COPD patients.

**Methods:**

This prospective, open-label, randomized controlled trial was registered in ClinicalTrials.gov: Identifier NCT05664347. After baseline assessment participants received either a verbal (control group) or a video-based (intervention group) TTG strategy. After 3-month the intervention was assessed for impact on the intended outcomes. Inhaler technique was assessed using standardized checklists, disease control using the Asthma control test and COPD assessment test respectively for asthma and COPD patients while adherence using the Morisky Green Levine scale. For QoL, the mini asthma quality of life questionnaire and the St. George respiratory questionnaire were used for asthmatic and COPD patients, respectively. Differences in outcomes between intervention-control groups were analyzed using either Chi-Square (X^2^)/Fisher Exact or Mann Whitney test. The impact of intervention on outcomes over-time was examined using either McNemar or Wilcoxon test.

**Results:**

At baseline, intervention (n = 51) and control (n = 52) groups had comparable demographic/clinical characteristics. At follow-up, inhaler technique improved among intervention group compared to control group (93.4% vs 67%) and to baseline (93.4% to 49.5%), (*P*<0.05). Similarly, medication adherence ameliorated among the intervention group in comparison to control group (88.2% to 61.5%) and to baseline (88.2% to 66.7%), (*P*<0.05). In regards to disease control, results showed an amelioration among the intervention group compared to baseline (35.3% to 54.9%) (*P*<0.05). QoL scores improved significantly among asthma patients (intervention group) at follow-up vs baseline. Better scores were also observed for COPD patients compared to controls, (*P*<0.05).

**Conclusion:**

Video-based (TTG) was effective in enhancing inhaler technique over time as well as improving disease control, medication adherence, and QoL.

**Trial registration:**

ClinicalTrials.gov: NCT05664347. https://clinicaltrials.gov/ct2/show/NCT05664347.

## Introduction

Asthma and COPD are two respiratory conditions affecting millions of people around the world leading to morbidity and mortality [1–8]. In Jordan, their prevalence is increasing with COPD still being largely underdiagnosed [9,10].

Inhaled medications are the cornerstone management for both asthma and COPD [1,2,11]. Despite mastery of inhaler technique is mandatory to control respiratory symptoms, up to 80% of asthmatic patients are unable to use their inhalers correctly and more than two-thirds of COPD patients make at least one error using their devices [1,2,12–14]. In Jordan, only around 40% of asthmatic adults demonstrated correct technique while no data is available for COPD patients [15]. Inhaler misuse has been associated with therapy failure, poor symptom control, lower quality of life (QoL), poor medication adherence and increased emergency department visits, hospitalizations, and use of oral corticosteroids [6,12,13,16–18].

Patient education on the correct use of inhalers can be crucial for technique mastery and the effective management of asthma and COPD, ameliorating symptom control, disease-related QoL, and medication adherence [16,19–21]. The teach-To-Goal (TTG) education is a multi-session educational approach that can be implicated to teach patients self-care skills and the proper use of medications, including inhalers, until they reach the learning goals (“Teach to Goal”) [22,23].

Despite being an effective educative method, studies showed that the inhaler technique, initially improved after a TTG education, deteriorates over time [23,24]. On these bases, patient education needs to be reinforced. Patients necessitate an effective, portable education that remains easily accessible whenever they need it. Technology-based interventions were shown to be effective at improving self-management of chronic conditions, including respiratory conditions [25–32]. However, no previous reports investigated the long-term effect of technology-based TTG education on patients with asthma and COPD. In Jordan, only few studies investigated the impact of inhaler technique education on asthma patients, none of these targeted COPD patients or implicated technology-based interventions [13,15,24,33,34].

The present study thus aimed to provide asthma and COPD patients with a novel inhaler educational approach, through the development of video-based Teach-To-Goal education that can be easily accessible by the patient at any time after the clinic visit. The study investigated the impact of this novel approach on the mastery of inhaler technique as well as other clinical parameters, asthma/COPD control, medication adherence, and quality of life.

## Methods

The protocol for this trial and the supporting Consort checklist are available as supporting information; see S1 Protocol and S1 Checklist.

### Settings and participants

A prospective, open label, randomized controlled trial was conducted at the respiratory clinics of Jordan University Hospital (JUH) and Islamic Hospital (IH) during the period between June/2020 and February/2021. All eligible participants were invited to take part in the study. Inclusion criteria included: 1) adult patients, 2) with established asthma or COPD diagnosis, 3) treated chronically with an inhaler (pressurized metered-dose inhaler (pMDI), Accuhaler, Respimat soft mist inhaler, Turbohaler, Handihaler, and/or Easyhaler), 4) since at least one month. Patients were excluded from the study if they were: 1) at high risk of infection (immunocompromised patients), 2) presenting with a very severe clinical presentation (severe dyspnoea, confusion due to hypoxemia, in need to continuous oxygen therapy).

### Ethics statement

Ethical approval was obtained from the institutional review boards of JUH (2020/15074) and IH (378/2021/1). A verbal consent was obtained from each participant before participation. The IRB committee approved the verbal consent procedure to avoid spread of COVID-19 virus from sharing papers and pens needed during the written consent procedure. The patients’ names, their phone numbers and the date of data collection were recorded on the data collection forms. All the data was appropriately coded and analyzed anonymously. Only eligible patients who approved to take part in the research and gave an informed consent were enrolled in the study.

**Fig 1:**
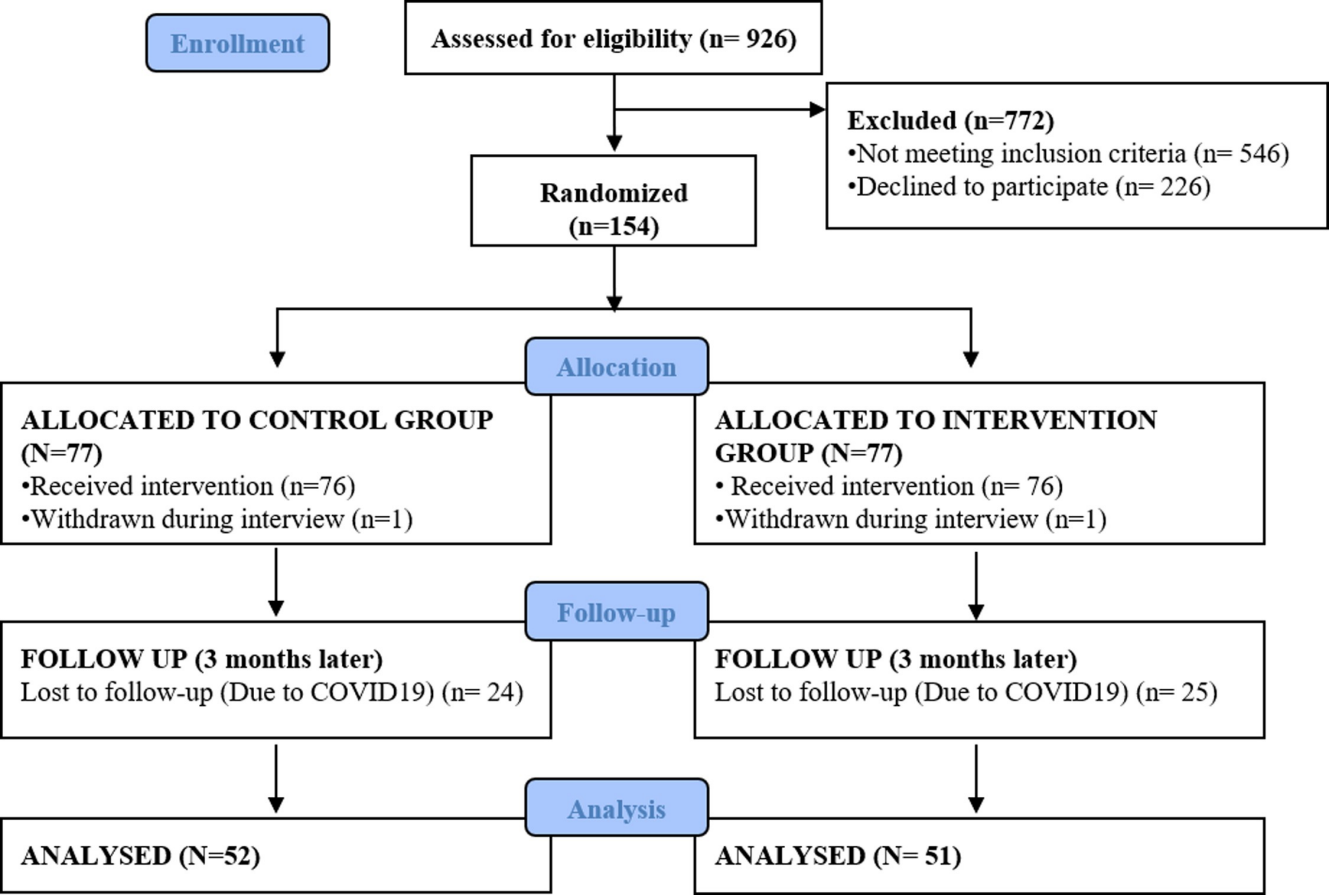
Consort flow diagram.

The patients, initially recruited by a clinical pharmacist in the clinic’s waiting room were asked to be interviewed in a separate place to assure privacy. To avoid spread of Corona virus, the clinical pharmacist was wearing a mask, gloves and a face shield during the whole interview. The trial was registered in the international trial register retrospectively [ClinicalTrials.gov: Identifier NCT05664347; 23rd December 2022]. The delay in the trial registration was due to investigators being unfamiliar to the administrative procedure of trials registration especially in the initial lack of a central institutional PRS administrator. The authors confirm that all ongoing and related trials for this intervention are registered. Fig 1 shows the Consort flow diagram.

### Randomization and the study outcomes

The study implicated meeting each patient at the time of recruitment (time zero) and after 3 months for follow-up. The enrolled participants were randomized using simple randomization into two groups; odd numbers for the control group and even numbers for the intervention group. The control group received a verbal teach-to-goal (TTG) inhaler technique education while the intervention group received a video-based TTG education.

**Fig 2:**
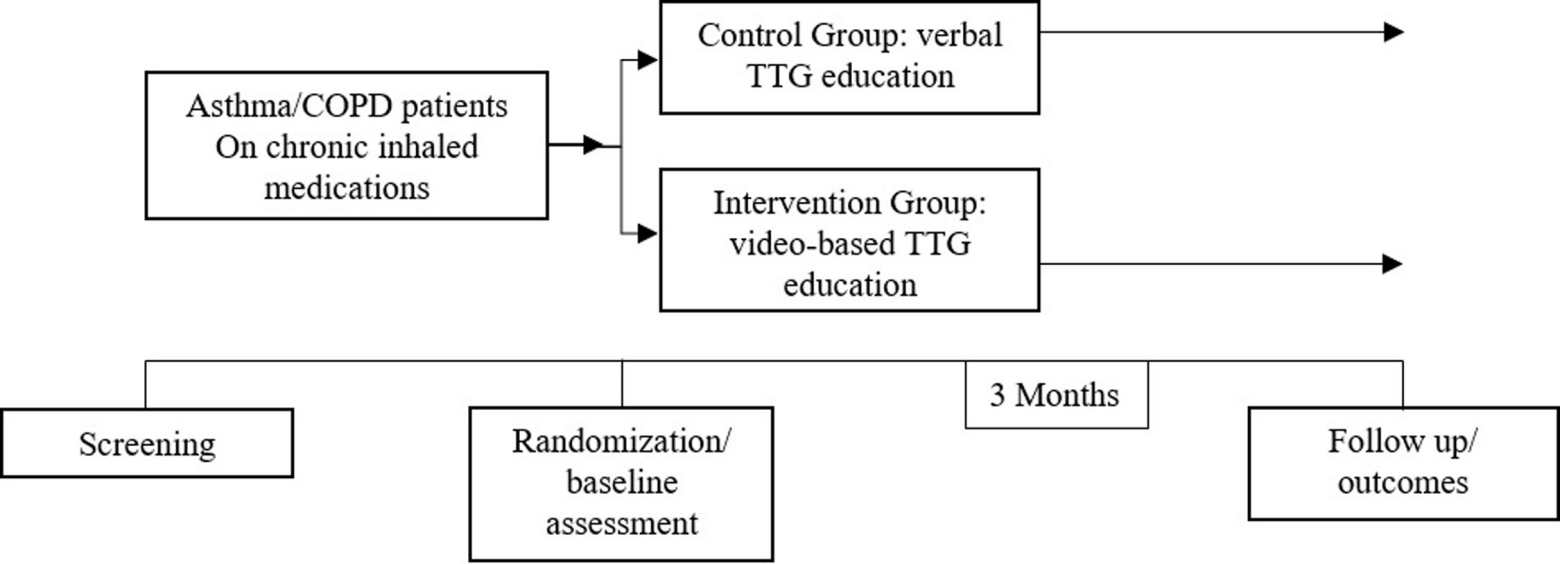
Schematic representation of the study design.

The main outcome of the study was inhaler technique mastery at follow-up (after 3 months). Secondary outcomes included amelioration of disease control, disease-related quality of life, and medication adherence at follow-up. Fig 2 shows a schematic representation of the study design.

### Data collection

A data collection form was used to collect information from the participants. A clinical pharmacist (research assistant) performed all the data collection. The data collection form comprised five parts; the first included patient demographics and clinical characteristics (e.g., age, gender, prescribed inhaled medications and comorbidities). The second part included checklists for the assessment of the inhaler technique. The inhaler checklists were obtained from the official websites of the inhaler-producing companies and were standardized to include 9 main steps for pMDI, Accuhaler, Turbohaler devices and 10 steps for Respimat, Breezhaler devices. All checklists are available in the (S1 File). Correct inhaler technique was defined as performing correctly 75% or more of the steps [23].

The third part of the data collection form assessed the patients’ disease control using the validated Arabic versions of the Asthma control test (ACT) and the COPD assessment test (CAT) for asthma and COPD patients respectively [1,2,35–37]. The ACT is a 5- items assessment tool with a score ranging between 5 and 25 where “higher scores indicate better control” [2,35]. The CAT is an 8-items assessment tool with a score ranging between 0 and 40 where “higher score indicates more burden and less disease control” [1,37].

The fourth part included the Arabic version of the 4-item Morisky Green Levine scale (MGLS) to assess medication adherence among the study participants [38]. MGLS has been used to assess medication adherence in various chronic diseases [39–42]. The scores were interpreted as follows: low adherence to the treatment plan (score 0–1) and moderate-high adherence (score 2–4).

Patients were considered to adherent to their medication if they were taking correctly all the prescribed controller inhalers. The fifth part assessed the disease-related QoL using the validated Arabic version of the mini asthma quality of life questionnaire (mini AQLQ) for asthmatic patients and the validated Arabic version of the St. George respiratory questionnaire (SGRQ) for COPD patients. Both tools, their domains and scoring systems were provided by email upon request by the developers [43,44].

The mini AQLQ is a 15-item questionnaire developed and validated by Juniper et al. (1999). It covers 4 domains (‘symptoms’, ‘activity limitations’, ‘emotional functions’, and ‘environmental stimuli’) with a score that ranges between 1 and 7 for each item. The 7-point scale is calculated by diving the total score (15 questions) by 7 to get a score that ranges from 1 to 7. Increase in the 7-point scale score indicates less impairment in the patient’s quality of life as described by [43].

The St George respiratory questionnaire (SGRQ) is a 50-item validated tool that covers three major domains: ‘symptom’, ‘activity’, and ‘impact’ [45]. Each question had a specific weight assigned by the developer and the SGRQ scores are calculated using an automated application also designed and afforded by the questionnaire developer [44].

The automated application can produce four scores; a total score, a ‘symptom’ score, an ‘activity limitation’ score and an ‘impact’ score. Each of these scores can range from 0 to 100, where a higher number indicates more impairment in the patient’s QoL [44]. The total QoL score, can be also categorized into quartiles; patients with a total SGRQ score of less than 32 were assigned to the 1^st^ quartile, while those with a score of 32–46, 46–60 and 60 or more were assigned to the 2^nd^ quartile, 3^rd^ quartile and fourth quartile, respectively [46].

### Educational tools and interventions

For each of the five types of inhalers, an educational video showing the correct inhaler technique was developed and recorded by the research team except for Breezhaler, for which the original company-producing video was used [47]. The videos were created to be clear, easy to understand, and in Arabic language. The educational videos showed all the steps necessary to master the inhaler technique as per the standardized checklists used to assess the inhaler technique. A link for each video is available in the (S1 File).

During the patients’ interview, the correct use of inhalers was assessed for each device using the respective checklist in order to obtain a baseline assessment of the patient’s inhaler technique (S1 File). Based on their randomization into control or intervention group, participants then received either a standard verbal TTG education or a video-based TTG educational intervention, respectively. The verbal TTG strategy (control) consisted of cycles of verbal demonstration and assessment, using the appropriate inhaler device, of the correct use of inhalers as per respective standardized checklist. On the other hand, the video-based TTG strategy (intervention) comprised cycles of demonstration and assessment of the correct inhaler techniques but using educational videos, that were displayed to patients through a smartphone. The videos showed step-by-step the correct inhaler technique as per standardized checklists. For each wrong step performed by the patient, the video was displayed again until technique mastering. At the end of the interview the intervention group received a copy of the video/s via WhatsApp^®^ and was invited to watch the video/s whenever needed.

### Follow up

After three months (follow-up interview), inhaler technique, disease control, medication adherence and disease-related quality of life were all assessed again.

For ethical considerations, at the end of the second interview, participants were encouraged to take their medications regularly as prescribed. In addition, they received a peak flow meter as compensation for taking part in the study.

### Data analysis and sample size

The targeted sample size was calculated using ClinCalc^®^ automated sample size calculator [48]. Based on published data, the prevalence of inhaler mastery among Jordanian adults with asthma is around 40% [15,33,34]. No data is available for COPD patients. Assuming an increase in the technique mastery from 40% to 80%, a statistical power of 90% and an alpha value of 0.05, the estimated sample size was 48 patients per study arm based on chi-square test of proportions.

All data were coded and analyzed using the statistical package SPSS (version 23). Sociodemographic and clinical characteristics were presented as frequency (percentage) for categorical variables and median (IQR) for continuous variables (variables were not normally distributed). Differences in the outcomes between the intervention and control groups (two independent groups) were assessed using Chi-Square (X^2^) Test or Fisher Exact Test for categorical/ordinal variables and Mann Whitney U test for continuous variables (variables were not normally distributed). The impact of the education on the inhaler technique, disease control, adherence and quality of life within each group over time (matched pairs) were examined using McNemar Test for categorical variables and Wilcoxon test for continuous/ordinal variables. *P*-value <0.05 indicated statistical significance.

## Results

A total of 926 patients were screened for participation in the study; 546 did not meet the inclusion criteria. Of those eligible to take part in the study, 228 refused to participate while the rest (152 patients) were enrolled in the study and randomized equally into control and intervention groups. No significant differences were initially observed between the two groups in their sociodemographic/clinical characteristics. After three months 103 patients (67%) returned for follow-up and their data included in the final analysis; 52 (50.5%) were in the control group (verbal TTG), 51 (49.5%) in the intervention group (video-based TTG). Fig 1 shows the Consort flow diagram.

No significant differences were observed in the sociodemographic/clinical characteristics between the patients included in the final analysis (patients followed up, N = 103) and those that dropped out from the study (N = 49).

### Patient demographic and clinical characteristics

Table 1 displays the baseline demographic and clinical characteristics of the participants (N = 103). Most of the study participants (N = 87, 84.5%) were asthmatic patients, the remaining (N = 16, 15.5%) had COPD.

**Table 1:** Participants’ baseline sociodemographic and clinical characteristics.

*Characteristic* ^*a*^	*All participants N = 103*	*Control group* *N = 52 (50*.*5%)*	*Intervention group* *N = 51 (49*.*5%)*	*P-value*
**Age** ^**b**^	55 (45–62)	54 (43–63)	55 (51–61)	0.754^d^
**Gender** Male Female	33 (32%) 70 (68%)	19 (36.5%) 33 (63.5%)	14 (27.5%) 37 (72.5%)	0.323^c^
**Marital status** Married Others	79 (76.7%) 24 (23.3%)	39 (75%) 13 (25%)	40 (78.4%) 11 (21.6%)	0.680^c^
**Education** Scholar degree Higher education	67 (65%) 36 (35%)	34 (65.4%) 18 (34.6%)	33 (64.7%) 18 (35.3%)	0.942^c^
**Occupation** Unemployed Employed Retired	43 (41.7%) 32 (31.1%) 28 (27.2%)	22 (42.3%) 17 (32.7%) 13 (25%)	21 (41.2%) 15 (29.4%) 15 (29.4%)	0.869^c^
**Monthly salary USD`** Less than 600 More than 600	70 (68%) 33 (32%)	33 (63.5%) 19 (36.5%)	37 (72.5%) 14 (27.5%)	0.323^c^
**Smoking status** Active smoker Nonsmoker Passive smoker	18 (17.5%) 42 (40.8%) 43 (41.7%)	10 (19.2%) 20 (38.5%) 22 (42.3%)	8 (15.7%) 22 (43.1%) 21 (41.2%)	0.847^c^
**Diagnosis** Asthma COPD	87 (84.5%) 16 (15.5%)	43 (82.7%) 9 (17.3)	44 (86.3%) 7 (13.7%)	0.616^c^
**Number of inhalers** 1 ≥2	38 (36.9%) 65 (63.1%)	20 (38.5%) 32 (61.5%)	18 (35.3%) 33 (64.7%)	0.739^c^
**Oral steroids last month** Yes No	27 (26.2%) 76 (73.8%)	14 (26.9%) 38 (73.1%)	13 (25.5%) 38 (74.5%)	0.869^c^
**Number of exacerbations last year** None or one without hospitalization One with hospitalization or more without	63 (61.2%) 40 (38.8%)	34 (65.3%) 18 (34.7%)	29 (56.9%) 22 (43.1%)	0.375^c^
**Number of hospitalizations last year** None ≥1	70 (68%) 33 (32%)	38 (73.1%) 14 (26.9%)	32 (62.7%) 19 (37.3%)	0.261^c^
**Vaccination status** None Flue Pneumococcal	88 (85.43%) 14 (13.6%) 1 (0.97%)	48 (92.3%) 4 (7.7%) 0 (0%)	40 (78.4%) 10 (19.6%) 1 (2%)	0.117^c^

^a^All data expressed as N (%) unless otherwise indicated.

^b^Data presented as median (IQR).

^c^Chi square test.

^d^Mann Whitney U test.

The median (IQR) age of all the participants was 55 [45–62) years. Most of the them were females (N = 70, 68%), married (N = 79, 76.7%), and had a scholarly degree (N = 67, 65%). The most were unemployed/retired (69%) and were receiving a salary of less than 600USD per month (N = 71, 68%). Of all the patients 17.5% (N = 18) were active smokers, 41.7% (N = 43) were passive smokers, and 40.8% (N = 42) nonsmokers.

More than half of the participants (N = 65, 63.1%) were prescribed two or more inhaler devices at the time of the recruitment with Turbohaler being the inhaler device mostly used among the study population (N = 58, 56.3%). Details regarding the number of participants using each type of inhaler are illustrated in the (S1 File).

Among all the patients, 38.8% (N = 40) had at least one acute exacerbation leading to hospitalization during the last year and 26.2% (N = 27) received oral corticosteroids during the previous month. When asked about vaccination, only 13.5% of the patients (N = 14) stated receiving annual influenza vaccine, just one reported being vaccinated against pneumococcus species.

When looking at the asthma and COPD comorbidities, allergic rhinitis (N = 61, 59.2%), and GERD (N = 45, 43.6%) were the two most prevalent conditions. More details are presented in the (S1 File).

No significant differences were observed in terms of demographic and clinical characteristics between the control and intervention groups at baseline.

### Inhaler technique

Inhaler techniques were assessed for each inhaler device used by each patient at baseline and at follow-up (after 3 months) using the checklists in the (S1 File). This resulted in a total of 182 assessments at baseline and similarly 182 at follow-up (91 per study arm).

Upon comparing inhaler technique at baseline (all the 182 assessments), 49.5% (N = 45) and 54.9% (N = 50) of the techniques resulted correct among the intervention and control group, respectively (*P* = 0.458) (Table 2). However, at follow up significant increase in the number of correct techniques was observed among the intervention group compared to control group (N = 85, 93.4% vs N = 61, 67%, *P* = 0.001) (Table 3).

**Table 2:** Baseline clinical outcomes of the study participants.

*Characteristic* ^*a*^	*All participants N = 103*	*Control group* *N = 52 (50*.*5%)*	*Intervention group* *N = 51 (49*.*5%)*	*P-value* *GLM*
**Inhaler technique score (all assessments)** Inappropriate technique Appropriate technique	87 (47.8%) 95 (52.2%)	41 (45.1%) 50 (54.9%)	46 (50.5%) 45 (49.5%)	0.458^c^
**pMDI technique score** Inappropriate technique Appropriate technique	21 (41.2%) 30 (58.8%)	10 (35.7%) 18 (64.3%)	11 (47.8%) 12 (52.2%)	0.382 ^c^
**Accuhaler technique score** Inappropriate technique Appropriate technique	21 (52.5%) 19 (47.5%)	10 (43.5%) 13 (56.5%)	11 (64.7%) 6 (35.3%)	0.184^c^
**Respimat technique score** Inappropriate technique Appropriate technique	21 (72.4%) 8 (27.6%)	12 (85.7%) 2 (14.3%)	9 (60%) 6 (40%)	0.215^f^
**Turbohaler technique score** Inappropriate technique Appropriate technique	23 (39.7%) 35 (60.3%)	8 (34.8%) 15 (65.2%)	15 (42.9%) 20 (57.1%)	0.539^c^
**Breezhaler technique score** Inappropriate technique Appropriate technique	1 (25%) 3 (75%)	1 (33.3%) 2 (66.7%)	0 (0%) 1 (100%)	1^f^
**Disease control (combined)** Uncontrolled symptoms Well-controlled symptoms	58 (56.3%) 45 (43.7%)	25 (48.1%) 27 (51.9%)	33 (64.7%) 18 (35.3%)	0.089^c^
**ACT scale**	19 (13–22)^b^	20 (14–23)^b^	17.5 (13–22)^b^	0.159^d^
**CAT scale**	13.5 (8.5–20)^b^	16 (8–23.5)^b^	13 (8–14)^b^	0.426^d^
**Adherence** Low adherence Moderate-High adherence	36 (35%) 67 (65%)	19 (36.5%) 33 (63.5%)	17 (33.3%) 34 (66.7%)	0.733^c^
**Total AQoL**	75 (62–85)^b^	79 (62–91)^b^	69.5 (54.5–83)^b^	**0.039** ^**d**^
**7-point scale AQoL** Total impairment Highly impaired Very impaired Moderate impairment Somehow impaired Slightly impaired No impairment	1 (1.1%) 3 (3.45%) 10 (11.5%) 18 (20.7%) 28 (32.2%) 21 (24.1%) 6 (6.9%)	0 (0%) 0 (0%) 5 (11.6%) 9 (20.9%) 13 (30.2%) 11 (25.6%) 5 (11.6%)	1 (2.3%) 3 (6.8%) 5 (11.4%) 9 (20.5%) 15 (24.1%) 10 (22.7%) 1 (2.3%)	0.335^c^
**Total symptoms AQoL**	24 (16–30)^b^	25 (18–32)^b^	21 (15–28)^b^	0.066^d^
**Total activity limitation AQoL**	21 (16–25)^b^	23 (18–26)^b^	19 (13.25–25)^b^	**0.029** ^**d**^
**Total emotional function AQoL**	20 (17–21)^b^	20 (18–21)^b^	19.5 (15–21)^b^	0.2^d^
**Total environmental stimuli AQoL**	10 (6–15)^b^	10 (8–14)^b^	10 (5.25–15)^b^	0.658^d^
**COPD Total QoL score**	39.55 (27.47–5)^b^	44 (28.8–46.8)^b^	32.3 (26.58–43.5) ^b^	0.186^d^
**COPD Total QoL (quartiles)** 1^st^ quartile 2^nd^ quartile 3^rd^ quartile 4^th^ quartile	5 (31.3%) 8 (50%) 3 (18.75%) 0 (0%)	2 (22.2%) 4 (44.4%) 3 (33.3%) 0 (0%)	3 (42.9%) 4 (57.1%) 0 (0%) 0 (0%)	0.223^c^
**COPD Symptoms QoL**	40.84 (25.2–50)^b^	40.31 (19.16–46.79) ^b^	47.1 (27.39–50.59) ^b^	0.368^d^
**COPD Activity QoL**	60.35 (47.69–72.98)^b^	72.29 (54–79.155) ^b^	54.54 (47.69–60.35) ^b^	0.071^d^
**COPD Impact QoL**	25.99 (14.2–29.6)^b^	26.09 (14.54–32.41) ^b^	15.1 (14.04–26.5) ^b^	0.315^d^

^a^All data expressed as N (%) unless otherwise indicated.

^b^Data presented as median (IQR).

^c^Chi square test.

^d^Mann Whitney U test.

^f^Fisher’s exact test.

Bold values indicate statistical significance *p*<0.05.

AQoL: Asthma Quality of Life.

**Table 3:** Follow up clinical outcomes of the study participants.

*Characteristic* ^*a*^	*Control group* *N = 52 (50*.*5%)*	*Intervention group* *N = 51 (49*.*5%)*	*P-value*
**Inhaler technique score (All assessments)** Inappropriate technique Appropriate technique	30 (33%) 61 (67%)	6 (6.6%) 85 (93.4%)	**<0.001** ^**c**^
**pMDI technique score** Inappropriate technique Appropriate technique	7 (25%) 21 (75%)	1 (4.3%) 22 (95.7%)	0.059^f^
**Accuhaler technique score** Inappropriate technique Appropriate technique	7 (30.4%) 16 (69.6%)	2 (11.8%) 15 (88.2%)	0.256^f^
**Respimat technique score** Inappropriate technique Appropriate technique	11 (78.6%) 3 (21.4%)	3 (20%) 12 (80%)	**0.002** ^**c**^
**Turbohaler technique score** Inappropriate technique Appropriate technique	4 (17.4%) 19 (82.6%)	0 (0%) 35 (100%)	**0.021** ^**f**^
**Breezhaler technique score** Inappropriate technique Appropriate technique	1 (33.3%) 2 (66.6%)	0 (0%) 1 (100%)	1^f^
**Disease control (combined)** Uncontrolled symptoms Well controlled symptoms	25 (48.1%) 27 (51.9%)	23 (45.1%) 28 (54.9%)	0.762^c^
**ACT scale**	20 (16–24)^b^	20 (17–22.75)^b^	0.912^d^
**CAT scale**	12 (3.5–24.5)^b^	8 (6–13)^b^	0.633^d^
**Adherence** Low adherence Moderate-High adherence	20 (38.5%) 32 (61.5%)	6 (11.8%) 45 (88.2%)	**0.002** ^**c**^
**Total AQoL**	80 (66–92) ^b^	78.5 (64.25–84.75) ^b^	0.405^d^
**7-point scale AQoL** Total impairment Highly impaired Very impaired Moderate impairment Somehow impaired Slightly impaired No impairment	0 (0%) 0 (0%) 2 (4.7%) 10 (23.3%) 13 (30.2%) 13 (30.2%) 5 (11.6%)	0 (0%) 1 (2.3%) 3 (6.8%) 9 (20.5%) 17 (38.6%) 12 (27.3%) 2 (4.5%)	0.684^c^
**Total symptoms AQoL**	27(20–33) ^b^	26 (21–30.75) ^b^	0.595^d^
**Total activity limitation AQoL**	21 (19–26) ^b^	21 (17.25–24) ^b^	0.261^d^
**Total emotional function AQoL**	20 (17–21) ^b^	21 (17.25–21) ^b^	0.858^d^
**Total environmental stimuli AQoL**	12 (7–15) ^b^	11 (9–14.75) ^b^	0.963^d^
**COPD Total QoL**	46.87 (27.55–51.19)^b^	28.5 (23.72–34.57)^b^	**0.039** ^**d**^
**COPD Total QoL (quartiles)** 1^st^ quartile 2^nd^ quartile 3^rd^ quartile 4^th^ quartile	2 (22.2%) 2 (22.2%) 4 (44.4%) 1 (11.1%)	5 (71.4%) 2 (28.6%) 0 (0%) 0 (0%)	0.105^c^
**COPD Symptoms QoL**	43.27 (20.97–54.66)^b^	41.48 (20.3–46.05)^b^	0.634^d^
**COPD Activity QoL**	72.29 (50.6–79.155) ^b^	47.71 (41.31–49.38)^b^	**0.039** ^**d**^
**COPD Impact QoL**	29.3 (16.61–36.87)^b^	17.76 (7.66–21.58)^b^	**0.044** ^**d**^

^a^ All data expressed as N (%) unless otherwise indicated.

^b^ Data presented as median (IQR).

^c^ Chi square test.

^d^ Mann Whitney U test.

^f^ Fisher’s exact test.

Bold values indicate statistical significance *P*<0.05.

AQoL: Asthma Quality of Life.

Technique mastery was also analyzed for each type of inhaler. No significant differences were detected in the number of patients mastering pMDI (*P* = 0.382), Accuhaler (*P* = 0.184), Respimat (*P* = 0.215), Turbohaler (*P* = 0.539) and Breezhaler (*P* = 1) between the control and intervention groups at baseline (*P*>0.05) (Table 2). At follow-up a significant difference was observed in the mastery of Respimat (*P* = 0.002) and Turbohaler (*P* = 0.021) in favor of the intervention group (Table 3).

When looking at the technique mastery over time within each group (Table 4), significant improvement was observed in all the assessments among the intervention group (from N = 45, 49.5% at baseline to N = 85, 93.4% at follow-up; P<0.001). Significant improvements were also observed in the numbers of patients mastering the technique of pMDI (from N = 12, 52.2% to N = 22, 95.7%; *P* = 0.002), Accuhaler (from N = 6, 35.3% to N = 15, 88.2%; *P* = 0.012) and Turbohaler (from N = 20, 57.1% to N = 35, 100%; *P*< 0.01). No significant differences were observed in the control group (*P*>0.05) (Table 4).

**Table 4:** Effect of interventions on participant’s clinical outcomes overtime.

*Characteristic* ^*a*^	*Control*			*Intervention*		
	*Baseline*	*Follow-up*	*P-value*	*Baseline*	*Follow-up*	*P-value*
**Inhaler technique (All assessments)** Inappropriate technique Appropriate technique	41 (45.1%) 50 (54.9%)	30 (33%) 61 (67%)	0.063^d^	46 (50.5%) 45 (49.5%)	6 (6.6%) 85 (93.4%)	**<0.001** ^**c**^
**pMDI technique** Inappropriate technique Appropriate technique	10 (35.7%) 18 (64.3%)	7 (25%) 21 (75%)	0.453^d^	11 (47.8%) 12 (52.2%)	1 (4.3%) 22 (95.7%)	**0.002** ^**c**^
**Accuhaler technique** Inappropriate technique Appropriate technique	10 (43.5%) 13 (56.5%)	7 (30.4%) 16 (69.6%)	0.549^d^	11 (64.7%) 6 (35.3%)	2 (11.8%) 15 (88.2%)	**0.012** ^**c**^
**Respimat technique** Inappropriate technique Appropriate technique	12 (85.7%) 2 (14.3%)	11 (78.6%) 3 (21.4%)	1^d^	9 (60%) 6 (40%)	3 (20%) 12 (80%)	0.070^c^
**Turbohaler technique** Inappropriate technique Appropriate technique	8 (34.8%) 15 (65.2%)	4 (17.4%) 19 (82.6%)	0.219^d^	15 (42.9%) 20 (57.1%)	0 (0%) 35 (100%)	**<0.001** ^**c**^
**Breezhaler technique** Inappropriate technique Appropriate technique	1 (33.3%) 2 (66.7%)	1 (33.3%) 2 (66.6%)	1^d^	0 (0%) 1 (100%)	0 (0%) 1 (100%)	-
**Disease control (combined)** Uncontrolled symptoms Well controlled symptoms	25 (48.1%) 27 (51.9%)	25 (48.1%) 27 (51.9%)	1^d^	33 (64.7%) 18 (35.3%)	23 (45.1%) 28 (54.9%)	**0.013** ^**c**^
**ACT scale**	20 (14–23)^b^	20 (16–24)^b^	0.18^g^	17.5 (13–22) ^b^	20 (17–22.75) ^b^	**<0.001** ^**d**^
**CAT scale**	16 (8–23.5) ^b^	12 (3.5–24.5) ^b^	0.292^g^	13 (8–14) ^b^	8 (6–13) ^b^	**0.033** ^**d**^
**Adherence** Low adherence Moderate-high adherence	19 (36.5%) 33 (63.5%)	20 (38.5%) 32 (61.5%)	1^d^	17 (33.3%) 34 (66.7%)	6 (11.8%) 45 (88.2%)	**0.003** ^**c**^
**Total AQoL**	79 (62–91) ^b^	80 (66–92) ^b^	0.164^g^	69.5 (54.5–83) ^b^	78.5 (64.25–84.75) ^b^	**<0.001** ^**d**^
**7-point scale AQoL** Total impairment Highly impaired Very impaired Moderate impairment Somehow impaired Slightly impaired No impairment	0 (0%) 0 (0%) 5 (11.6%) 9 (20.9%) 13 (30.2%) 11 (25.6%) 5 (11.6%)	0 (0%) 0 (0%) 2 (4.7%) 10 (23.3%) 13 (30.2%) 13 (30.2%) 5 (11.6%)	0.176^g^	1 (2.3%) 3 (6.8%) 5 (11.4%) 9 (20.5%) 15 (24.1%) 10 (22.7%) 1 (2.3%)	0 (0%) 1 (2.3%) 3 (6.8%) 9 (20.5%) 17 (38.6%) 12 (27.3%) 2 (4.5%)	**0.001** ^**d**^
**Total symptoms AQoL**	25 (18–32) ^b^	27(20–33) ^b^	0.243^g^	21 (15–28) ^b^	26 (21–30.75) ^b^	**<0.001** ^**d**^
**Total activity limitation AQoL**	23 (18–26) ^b^	21 (19–26) ^b^	0.670^g^	19 (13.25–25)^b^	21 (17.25–24) ^b^	**0.004** ^**d**^
**Total emotional function AQoL**	20 (18–21)^b^	20 (17–21) ^b^	0.987^g^	19.5 (15–21)^b^	21 (17.25–21) ^b^	**0.018** ^**d**^
**Total environmental stimuli AQoL**	10 (8–14) ^b^	12 (7–15) ^b^	0.472^g^	10 (5.25–15) ^b^	11 (9–14.75) ^b^	0.126^d^
**COPD Total QoL**	44 (28.8–46.8) ^b^	46.87 (27.55–51.19)^b^	0.674^g^	32.3 (26.58–43.5) ^b^	28.5 (23.72–34.57)^b^	0.128^d^
**COPD Total QoL (quartiles)** 1^st^ quartile 2^nd^ quartile 3^rd^ quartile 4^th^ quartile	2 (22.2%) 4 (44.4%) 3 (33.3%) 0 (0%)	2 (22.2%) 2 (22.2%) 4 (44.4%) 1 (11.1%)	0.18^g^	3 (42.9%) 4 (57.1%) 0 (0%) 0 (0%)	5 (71.4%) 2 (28.6%) 0 (0%) 0 (0%)	0.317^d^
**COPD Symptoms QoL**	40.31 (19.16–46.79) ^b^	43.27 (20.97–54.66)^b^	0.866^g^	47.1 (27.39–50.59) ^b^	41.48 (20.3–46.05)^b^	**0.046** ^**d**^
**COPD Activity QoL**	72.29 (54–79.155) ^b^	72.29 (50.6–79.155) ^b^	0.715^g^	54.54 (47.69–60.35) ^b^	47.71 (41.31–49.38)^b^	**0.046** ^**d**^
**COPD Impact QoL**	26.09 (14.54–32.41) ^b^	29.3 (16.61–36.87)^b^	0.173^g^	15.1 (14.04–26.5) ^b^	17.76 (7.66–21.58)^b^	0.463^d^

^a^ All data expressed as N (%) unless otherwise indicated.

^b^ Data presented as median (IQR).

^c^ McNemar test.

^d^ Wilcoxon test.

Bold values indicate statistical significance *P*<0.05.

AQoL: Asthma Quality of Life.

### Disease control

At baseline, of the 103 participants 43.7% (N = 45) had a well-controlled, less symptomatic condition (Table 2). No significant differences were detected between the intervention and control group in terms of disease control neither at baseline (Table 2) nor at follow-up (Table 3).

Over time, however, more participants in the intervention group had a well-controlled, less symptomatic condition compared to baseline (N = 28, 54.9% at follow-up vs N = 18, 35.3% at baseline; *P* = 0.013). When looking at the ACT and CAT scores, significant ameliorations were also observed in the median (IQR) of both scores over time within the intervention group (*P*<0.05) compared to baseline (Table 4).

### Medication adherence

Among all the 103 participants, 65% (N = 67) of them had a moderate-high level of adherence at baseline, with no significant difference observed between the control and intervention groups at baseline (*P* = 0.733) (Table 2). Interestingly, at follow-up, a significant difference was observed between the two groups (N = 45, 88.2% of the intervention group were adherent vs N = 32, 61.5% of the controls; *P*-value = 0.002). (Table 3).

Significant amelioration in the medication adherence was also detected within the intervention group over time, with 88.2% (N = 45) showing moderate-to-high adherence at follow-up compared to 66.7% (N = 34) at baseline (p = 0.003) (Table 4).

### Disease-related quality of life

When looking at the disease-related QoL, the total AQoL and the activity limitation AQoL scores were initially significantly different between the two groups at baseline, with the control group showing higher scores (i.e., better quality of life) (*P* = 0.039) (Table 2). Three months later, these differences were not anymore detected and the intervention group showed a non-significant amelioration in both scores (Table 3).

Significant ameliorations were also observed overtime in the total (*P* <0.001), symptom (*P* = 0.001), activity limitation (*P* = 0.004) and emotional function (*P* = 0.018) AQoL scores within the intervention group in comparison to baseline. This group also showed less impaired QoL over time as per the 7-point scale AQoL (*P* = 0.001) (Table 4).

In regards to COPD patients, no differences were detected in the QoL between the control and intervention groups at baseline (Table 2), however, at follow-up a significant p-value was observed in the total QoL score as well as the activity and impact QoL scores (intervention vs control) (Table 3). Over time, significant ameliorations in the symptom (*P* = 0.046) and activity scores (*P* = 0.046) were detected within the intervention group at follow-up in comparison to baseline (Table 4).

## Discussion

This is the first RCT investigating the effect of a video-based teach-to-goal educational intervention on inhaler technique mastery, in addition to its effect on disease control, disease-related quality of life, and medication adherence among adults with asthma and COPD over a period of three months.

The underdiagnosis of COPD among Jordanians might have contributed to the discrepancy in the number of asthma and COPD patients enrolled in the study [10,49,50]. In addition, most of the encountered COPD patients didn’t meet the inclusion criteria (for instance, they were critically ill). Assessment of the participants’ demographic characteristics showed consistency with the literature with predominance of male and female gender among COPD and asthma patients respectively [1,2].

Upon assessment of all the inhaler techniques used by the study population, almost half of the techniques were incorrect at baseline. This result cannot be compared to previously published data due to the presence of several checklists and scoring systems used to evaluate the correct use of inhalers [51]. However, worldwide studies, including Jordan reported that 29–94% of the patients use their inhalers incorrectly [11,13,23,24,32–34,52–57].

Turbohaler was the inhaler most frequently prescribed among the study participants which goes in line with the GINA recommendations to prescribe formoterol/budesonide combination (Turbohaler-Symbicort^®^) as the maintenance and reliever therapy for the treatment of asthma. The widespread and longtime use of Turbohaler might have contributed to the device being the most correctly used among the study participants.

The educational intervention adopted in this study demonstrated superiority over the verbal TTG education and it was able to retain good technique over time. The uniqueness of this intervention is that it blends the initial face-to-face education with a technology-based education. This pinpoints the advantage of interaction with the clinical pharmacists in addition to that offered by a video [29]. Video-based educations have the ability to simulate physical demonstration that if added to verbal and/or written instructions can result in device technique improvement [56]. In addition, when supported by social media, videos have the advantage to be easily accessible, well-reached, and free of charge allowing patients to reinforce their knowledge after the initial education [31].

Interestingly, when Press et al. (2017) studied the effectiveness of a virtual TTG education without providing direct contact with healthcare providers, patients showed good theoretical knowledge but incorrect inhaler techniques at the time of education [32]. Previous literature also revealed insufficiency of one TTG session provided directly by health care providers on the long-term retention of technique mastery [23,29,33,58,59]. During the clinic visit, patients might be tired and frustrated, which if added to limited time and knowledge of healthcare providers may let to provision of unsatisfactory education [11,33,60,61]. On this basis, patients need multiple sessions of education to fully understand (master) the inhaler technique [62,63]. These suggest that hybridization of face-to-face education to virtual one is needed to retain the correct technique over time. In this line, Bosnic-Anticevich et. al (2010) showed the addition of physical demonstration to written and verbal instructions to be associated with improvement in pMDI technique [56]. Similarly, van der Palen *et al*. found patients receiving group education on inhaler technique, and those receiving video-based instructions in addition to a home-taken videotape, having better ability to retain inhaler mastery over up to 9 months compared to those receiving classical in-person education [29].

When looking at disease control, the non-significant amelioration between the intervention and control groups at follow-up can result from the initial baseline difference observed in the ACT and CAT levels in favor to the control group. Significant amelioration was however clearly observed after three months within the intervention group in comparison to baseline, which might be a result of inhaler technique improvement among these participants. Mastery of inhaler technique has, in fact, been directly correlated to better control of disease-related symptoms such as shortness of breath and cough, as well as fewer exacerbations. [1,2,12,17,64–66]. For instance, a literature review by Usmani et al. (2018) reported higher number of inhaler errors associated with poorer disease outcomes and similarly, positive correlation between inhaler technique education, technique mastery, and disease control among asthma and COPD patients [17]. These results go in line with the recommendations from GINA and GOLD guidelines, stressing on the importance of inhaler technique mastery to improve disease control and reduce exacerbation risk [1,2].

Improvement in the symptoms and activity domains of the disease-related QoL might also be attributed to the amelioration of inhaler technique and its subsequent benefit on disease control among the intervention group [67,68]. As showed by different studies better disease control is positively correlated to better quality of life among asthmatic and COPD patients [68–72].

Medication adherence was also improved significantly over time among participants receiving the video-based TTG education. Better understanding the device technique should have contributed to patients feeling more comfortable in using their inhalers improving adherence [20,21,65,73,74]. On these bases, Ovchinicova et al. (2011) observed positive relationships between disease control, retaining good inhaler technique and medication adherence. Patients feeling improvement with treatment, were more motivated to use their inhalers correctly in terms of dosage regimen and technique [58]. Similar effect was also detected by Takemura et al. demonstrating significant impact of medication adherence on disease-related quality of life among patients with asthma and COPD [20,21].

Study limitations include the loss of some patients at follow-up. Due to the COVID-19 pandemic some participants preferred not to visit the study site. Moreover, despite the intention to address both asthma and COPD, the study didn’t achieve a balance in the number of participants with a dominance of asthmatic patients.

In conclusion, this study demonstrates that the video-based TTG education could significantly improve the inhaler technique mastery, disease control, disease-related patients’ quality of life and the medication adherence among patients with chronic respiratory conditions. The long-term effect on the study secondary outcomes is suggested to be directly related to the improvement in the inhaler technique mastery and to the reinforcing nature of the videos, easily accessible by the patients at any time.

## Supporting information

S1 Checklist Consort checklist CONSORT 2010 checklist of information to include when reporting a randomised trial*. DOI: 10.6084/m9.figshare.22133342.
(DOC)

S1 Protocol Study protocol. DOI: 10.6084/m9.figshare.22133306.
(DOC)

S1 File Supplementary methodology and results. DOI: 10.6084/m9.figshare.22133276.
(DOCX)
