# Supplementary material for: Video-based teach-to-goal intervention on inhaler technique on adults with asthma and COPD: A randomized controlled trial
Source: PLoS One. 2023 Jun 9;18(6):e0286870. doi: 10.1371/journal.pone.0286870 (PMC10256228; doi:10.1371/journal.pone.0286870)
Supplement: S1 Protocol — DOI: 10.6084/m9.figshare.22133306. (DOC) [file pone.0286870.s002.doc]

**STUDY PROTOCOL S1.**

**STUDY TITLE**

Implementing patient education on inhaler technique through a video-based teach-to-goal intervention and its impact among Jordanian adults with asthma and COPD.

**INTRODUCTION**

Asthma and COPD are two respiratory conditions characterized by chronic inflammation of the lungs resulting in respiratory impairment and distress (1, 2). They are considered a serious global problem affecting millions of people all over the world (3, 4).

The delivery of drugs by inhalation is an integral component for the treatment and management of respiratory conditions. Inhaled medicines are rapidly directed to the airways, allowing for rapid onset which permits the administration of lower doses, limiting potential side effects (5).

Inhaled corticosteroids (ICS) and bronchodilators, given on a regular basis, are considered central to symptoms management in asthma and COPD respectively and decrease in their exacerbation rates (1, 2).

The correct use of inhalers requires special techniques that are crucial for the optimal drug delivery and deposition in its site of action and considered mandatory to control respiratory symptoms (6-8). Data shows however, that inhaler mishandling remains a common problem in real life (6). Worldwide up to 80% of people with asthma are unable to use their inhaler device correctly and more than two thirds of patients with COPD make at least one error in using their inhalation device (1, 2).In Jordan, only 35% of asthmatic patients demonstrated correct technique while no data is available for COPD patients (9).

Inhaler misuse has been associated with poor symptom control and disease instability, increased risk of emergency visits and hospitalizations, use of oral corticosteroids, lower quality of life (QoL) and greater health-economic burden (5-7). Educating patients on how to use their inhaler correctly is thus considered crucial for the mastery of good inhaler technique and effective self-management of asthma and COPD (1, 2, 6).

The British thoracic society highlights the necessity of proper inhaler technique: “prescribe inhalers only after patients have received training in the use of the device and have demonstrated satisfactory technique” (10, 11).The Global Initiative for Asthma (GINA) and Global Initiative for Chronic Obstructive Lung Disease (GOLD) also stress to assess and correct poor inhalation technique before stepping up asthma or COPD treatment in patients with uncontrolled condition (1, 2).

The Teach-To-Goal (TTG) approach is considered an effective method to educate patients on the proper use of medications, including inhalers. It consists in in-person oral instructions using an inhaler device with cycles of demonstration and assessment (12, 13). However, after the initial education provided upon inhaler prescription, only few patients later receive information or advice on how to use their inhaler. Inhaler technique deteriorates over time; the improved inhaler technique initially obtained after TTG education wanes within 30 days post-discharge (13, 14).

On these bases, patient education needs to be reinforced. Patients necessitate an effective, portable education that remains easily accessible to them whenever they need it.

Few previous studies have shown technology-based interventions to be effective at improving self-management of few chronic conditions, including asthma however, none of these studies has been conducted in Jordan (15-19).

The present project thus aims to provide asthma and COPD patients in Jordan with a novel inhaler education approach, through the development of a Teach-To-Goal video-based education in Arabic that would be easily accessible post-discharge from patients’ home. The impact of this novel approach on inhaler technique as well as other clinical parameters such as asthma/COPD control, adherence to inhaler, and quality of life will also be assessed.

**Objectives:**

The objectives of the present research proposal are:

- To assess inhaler techniques, level of asthma/COPD control, medication adherence, quality of life and asthma- and COPD-related knowledge among Jordanian patients with asthma or COPD.
- To evaluate and compare the effectiveness of two methods of patient education on mastering inhaler technique among Jordanian patients with asthma and COPD; the traditional in-person TTG education and the video-based TTG one. This will be done immediately after the intervention and three months later.
- Assess the impact of the two interventions at the level of asthma and COPD control, medication adherence and quality of life on Jordanian patients with asthma and COPD after 3 months of the intervention.
- To evaluate the impact of the video-based TTG patient education on asthmatic and COPD patients with low literacy in comparison to high literacy patients.

**LITERATURE REVIEW**

Asthma and COPD, two chronic inflammatory conditions of the lungs, affect millions of people all over the world (WHO). Their prevalence is increasing in Jordan and COPD is still largely underdiagnosed and undertreated (20, 21).

Medications delivered through inhaler devices are the mainstay therapy for respiratory conditions (5).Inhaler devices require proper technique crucial for the optimal drug delivery and deposition in its site of action for both controller and reliever (rescue) medications (22). This however needs multiple, complex steps, some of which considered critical to the efficacy of the inhaled treatment, making inhaler devices difficult to use (5, 13).

Incorrect inhaler technique has been reported worldwide in asthma and COPD patients for pressurized metered dose inhalers and dry powder inhalers, with most of these patients being even not aware of having problems is using their device (1, 2, 23).In a recent study conducted in Jordan, only 35% of asthmatic patients demonstrated proper technique, while no studies investigated inhaler misuse in patients with COPD (9).

Poor technique has been associated with increased frequency of inhaler use and frequency of emergency department visits indicating a poorly controlled condition. It has also been associated with higher local and/or systemic side-effects, higher risk of exacerbation, increased use of oral corticosteroid and decreased quality of life in patients with asthma and COPD (22). In Jordan, a cross-sectional study showed that only one-third of asthmatic patients has controlled asthma while no data is available for COPD patients (24).

The GINA, GOLD and the British guidelines strongly address the importance of patient education on inhaler technique, considered crucial for the mastery of good inhaler technique and effective self-management of asthma and COPD (1, 2, 6, 10, 11). Despite its importance, suboptimal education has been reported (6).

Several are the studies that investigated the effect of patient education on the proper use of inhalers and disease control in asthma and COPD (25, 26).In particular, the Teach-To-Goal (TTG) intervention has been demonstrated to be more effective than the standard education usually provided to the patients at hospitals (single brief set of verbal step-by-step instructions) in improving inhaler technique, particularly for those with low health literacy (12, 13, 19, 25, 27).

Teach‐to‐goal (TTG) education is a multi-session patient education used to teach patients self-care skills until they reach behavioural goals (12, 28). It is based on testing effect and allowing memory to be enhanced through the act of retrieving information while learning. It employs initial assessment, instruction, and reassessment with repeated rounds of instruction until mastery is confirmed (12).

Patient education also revealed a better health status in those receiving the intervention reported as higher level of asthma/COPD control, less emergency visits, and lower risk of exacerbations (29-31).

Similarly, in Jordan an inhaler technique training delivered by pharmacist through “show-and-tell” approach enhanced inhaler technique and asthma control in hospitalized asthmatic patients (9).Similar results were also observed in patients with asthma in rural areas in Jordan (14). Two different novel approaches of education using inhaler reminder labels and pharmaceutical pictogram, were also recently investigated in Jordan, demonstrating better inhaler technique in patient with asthma (32, 33).The reminder labels, but not pharmaceutical pictograms also improved asthma control (32, 34). No previous studies have however assessed the relationship between patient education and proper inhaler technique is COPD patients in Jordan.

The positive impact of patient education is not limited to the acquisition of proper device technique but it was also showed to improve adherence to medications in asthma and COPD as well as the patients’ quality of life. In fact, correct use of inhalers is considered an important component in adherence and significantly correlated with a higher health-related quality of life score (6, 29, 30, 35).

Beside the positive impact of the different educational interventions, the best and most effective method to deliver such training to improve inhaler technique and clinical outcomes, remains unclear (25, 31).

The improved inhaler technique obtained after TTG education wanes within 30 days post-discharge. After the initial education provided upon inhaler prescription, only few patients later receive information or advice on how to use their devices and the inhaler technique deteriorates over time (13, 14).Thus, patient education needs to be reinforced; patients necessitate repeated instructions on inhalation technique to maintain long-term proper technique.

With increasing internet access among the population worldwide, technology-based interventions have been implemented and deemed effective alternatives to improve self-management of chronic conditions, including asthma (15-19). Previous studies showed that video-based intervention was more effective than written instruction or in-person TTG education in improving inhaler technique and main (18, 36).

Video-based instruction is thus considered an effective portable education intervention that remains accessible to patients for reinforcement of proper inhaler technique whenever needed.

**METHODOLOGY**

**Study design and participants:**

A prospective, open label, randomized controlled trial will be conducted at the Jordan University Hospital (JUH) in cooperation with Dr. Natheer obaidat from the respiratory team at (JUH), the Jordan Hospital and others public hospitals. An ethical approval will be obtained from the institutional review boards of the JUH, the Jordan Hospital and the Ministry of Health before starting the study.

All eligible patients between 17 and 80 years old attending the respiratory outpatient or the inpatient respiratory clinic in the hospitals will be invited to join the study and asked to sign a consent form before their participation.

Inclusion criteria include patients with an established asthma or COPD diagnosis by a respiratory physician, who were started on inhaler/s (pressurized metered dose inhaler (pMDI), turbohaler, accuhaler, handihaler, and soft mist inhaler) for a minimum of one month and are expected (as prescribed) to continue using their inhalers for a minimum of three months. Immunocompromised patients, patients who have difficulty in communication due to special needs or very severe clinical presentation will be excluded from the study.

Participants will be randomized into two groups. Simple randomization will be adopted; odd number for the educational intervention and even number for control.

Later each group will be provided, by a clinical pharmacist, with a different inhaler technique educational intervention (Non-invasive). The control group patients will receive an in-person TTG education consisting of a brief counseling on the proper use of inhalers followed by assessment. This cycle of assessment and counseling will be repeated up to three times if necessary, until the patient demonstrated correct technique on all steps.

Patients in the intervention group will receive a video-based teach-to-goal intervention on the proper inhaler technique using videos provided by the originator company of each inhaler type. Patients will be asked to watch a video (3-5 minutes in length) followed by assessment. If the assessment reveals incorrect use of the inhaler, the participants will be prompted to watch the video again and repeat until mastery will be demonstrated. If needed, the correct answer will be provided following 3 incorrect attempts. Patients in this group will be provided the video as a multimedia file to access it whenever they need re-education regarding their inhaler technique.

Based on published data, around 40% of Jordanian patients demonstrated proper inhaler technique after 3 months they have been provided the “Show-and-Tell” intervention, which is very similar to the TTG intervention (9). Thus, in order to detect 80% increase in the number of patients with correct use of inhaler, and accounting for 20% dropouts during the study period a sample size of 72 participants is required in each group at 95% statistical power and 5% significance level (Clincal.com).

**Baseline assessment and outcomes:**

At baseline, data regarding demographics, asthma/COPD history, medication use including adverse effects, hospital admissions, and previous systemic corticosteroid use.

Inhaler technique as well as the level of asthma/COPD control, adherence to medication and the patients’ quality of life will be assessed at baseline.

Initially, all participants will be asked to use their inhaler in front of the clinical pharmacist and the proper technique will be assessed by the aid of standardized device-specific inhaler technique checklists (AstraZeneca Pharmaceuticals, GlaxoSmithKline, Boehringer Ingelheim Pharmaceuticals) translated into Arabic. Each checklist includes 9-10 steps, eight of them considered essential for the medication to reach the site of action. A score of 8/10 will be thus considered and classified as correct inhaler technique for all of them.

All participants will be assessed for their level of asthma or COPD control through validated Arabic version of the Asthma Control Test )ACT( or COPD assessment test (CAT) before intervention (38, 39).

Medication adherence and quality of life will be assessed by the Arabic versions of the validated 8-items Morisky medication adherence scale (MMAS) of the St. George’s respiratory questionnaire respectively before and after intervention (2, 40-42).

The primary outcome for this research project is to improve inhaler technique in patients with asthma and COPD. Secondary outcomes include increase the level of asthma and COPD control, improve medication adherence, and quality of life.

After collection of all information and assessment of parameters at baseline, patients will receive either the in-person education or the video-based teach-to-goal intervention. Participants will be then asked to use their inhaler as prescribed by the physician.

All patients will be followed up for 3 months, after which inhaler technique, level of asthma/ COPD control, adherence to medication and the patients’ quality of life will be assessed.

**Analysis**

All data will be coded and entered to statistical software SPSS (version 23). Continuous variables will be presented as mean ± standard deviation while categorical variables will be presented as counts and percentages. The difference in the responses between the two groups will be examined using unpaired test t-test or Mann-Whitney U test for continuous variables and Chi-square (X^2^) test or Fisher’s exact test for categorical variables. The impact of the education on the inhaler technique, disease control, quality of life, and adherence within each group over time were examined using McNemar Test for categorical variables and Wilcoxon test for continuous/ordinal variables. All tests will be two-sided and statistical significance will be set at *p*-value ≤ 0.05. Intention to treat analysis will be adopted to handle missing data.

**REFERENCES**
