# Supplementary material for: Video-based teach-to-goal intervention on inhaler technique on adults with asthma and COPD: A randomized controlled trial
Source: PLoS One. 2023 Jun 9;18(6):e0286870. doi: 10.1371/journal.pone.0286870 (PMC10256228; doi:10.1371/journal.pone.0286870)
Supplement: S1 File — DOI: 10.6084/m9.figshare.22133276. (DOCX) [file pone.0286870.s003.docx]

**Supplementary methodology and results S1**

| Skill | |
| --- | --- |
|  | Remove cap from inhaler and spacer |
|  | Shake inhaler well |
|  | Hold inhaler upright |
|  | Breath out completely away from the inhaler |
|  | Close lips around mouth piece |
|  | Activate inhaler by pressing the canister once and take a deep slow breath, no whistle sound. |
|  | Breath in simultaneously with activation |
|  | Hold breath for five seconds while removing the MDI from your mouth then breath normally |
|  | Breath normally for 30-60 seconds, Repeat the sequence for second inhalation |

**Supplementary methodology:**

**Table S1. pMDI Checklist**

Ref: Press et al., 2017 [1]

**Table S2. Accuhaler Checklist**

|  | Skill |
| --- | --- |
| 1 | Open the accuhaler by pushing the thumb grip right around until it clicks. |
| 2 | Keep inhaler horizontally |
| 3 | Push the lever away until it stops |
| 4 | Note the decrease in the number on the dose counter |
| 5 | Breath out as much as possible away from the inhaler |
| 6 | Put the mouth piece between your lips and take a strong deep steady breath |
| 7 | Remove the accuhaler and hold breath for 10 seconds then breath out slowly |
| 8 | Close the accuhaler by sliding the thumb grip back to its original position |
| 9 | Rinse your mouth and gargle with water after taking your dose |

Ref: Seretide official website[2]

**Table S3. Respimat Checklist**

|  | **Spiriva Respimat: Preparation for use** |
| --- | --- |
|  | Skill |
|  | • Press the safety catch button while firmly pulling the clear base straight off with your other hand. Be careful not to touch the piercing element |
|  | Insert the narrow end of your medication cartridge into the inhaler. Place the inhaler on a flat surface, and push down firmly until it clicks into place |
|  | Replace the clear base by aligning the notch with the safety catch button, and slide together until it clicks |
|  | **Spiriva Respimat: How to prime** |
|  | Skill |
|  | With the cap closed, turn the clear base in the direction of the arrows on the label until it clicks (half a turn) |
|  | Push up on the small, circular opening tab until the cap snaps fully open |
|  | With the inhaler pointed toward the ground, press the dose release button. Look for a mist, repeat steps 3 & 4 until 4 visible mists have been dispensed. |
| **Spiriva Respimat: How to Use** | |
|  | Skill |
|  | With the cap closed, turn the clear base in the direction of the arrows on the label until it clicks (half a turn), then Open the hinged cap by pushing up on the small, circular tab until the cap snaps fully open |
|  | Breathe out slowly and fully. Close your lips around the mouthpiece without covering the air vents |
|  | Point the inhaler to the back of your throat. While taking a slow, deep breath through your mouth, press the dark gray doserelease button and continue to breathe in |
|  | Hold your breath for 10 seconds or for as long as comfortable |

Ref: GmbH official website [3]

**Table S4. Turbohaler Checklist**

|  | Skill |
| --- | --- |
| 1 | Unscrew and remove the cover |
| 2 | Understand that dose counter moves every 20 doses |
| 3 | Keep inhaler upright |
| 4 | Twist grip at the base (twist right and left until you hear a click) |
| 5 | Breath out gently away from inhaler |
| 6 | Place mouth piece between your teeth (don’t bite), close lips and take a strong deep steady breath |
| 7 | Remove the inhaler from your mouth and hold for 10 seconds then breath out gently |
| 8 | Replace cover |
| 9 | Rinse your mouth and gargle with water after taking your dose |

Ref: National asthma counsel Australia[4]

**Table S5. Breezhaler checklist**

|  | Skill |
| --- | --- |
| 1 | Remove the cap and |
| 2 | 1. Tilt the mouthpiece to open. |
| 3 | 1. Remove capsule from blister and place in chamber. |
| 4 | 1. Close mouthpiece until it clicks. |
| 5 | 1. Press side piercing buttons in once and release. (Do not shake.) |
| 6 | 1. Breathe out gently (away from inhaler). |
| 7 | 1. Put mouthpiece between teeth (without biting) and close lips to form good seal Breathe in rapidly and steadily, so capsule vibrates Continue to breathe in as long as comfortable. |
| 8 | 1. Hold breath for about 5 seconds, or as long as comfortable. While holding breath, remove inhaler from mouth. |
| 9 | 1. Breathe out gently (away from inhaler). |
| 10 | 1. Open mouthpiece and remove used capsule. If more than one dose is needed,* repeat all steps starting from step 3. |
| 11 | 1. Close mouthpiece and cap |

Ref: National asthma counsel Australia.

**Table S6. Inhaler technique educational videos**

| **Inhaler type** | **Video Link** |
| --- | --- |
| pMDI | https://www.youtube.com/watch?v=uLh4Vthv5vA&t=15s |
| Accuhaler | https://www.youtube.com/watch?v=eg6Tkh3EwG0&t=32s |
| Respimat | https://www.youtube.com/watch?v=eVTVPZGmg6o&t=31s |
| Turbohaler | https://www.youtube.com/watch?v=coJWqrrqolw&t=57s |
| Breezhaler | <https://www.youtube.com/watch?v=P16vS8_4HqE> |

**References**

1. Press, V.G., et al., *Virtual Teach-To-Goal Adaptive Learning of Inhaler Technique for Inpatients with Asthma or COPD.* J Allergy Clin Immunol Pract, 2017. **5**(4): p. 1032-1039.e1.

2. GSK. *How to use Seretide® Diskus®*. 2016 2016, April 2019, October 28]; Available from: <https://gskpro.com/content/dam/global/hcpportal/en_IE/Images/Seretide/IE_SFC_0007_15_Seritade_LP_GSKDC-PT-IRL-2016-9101_D1_Highres.pdf>.

3. GmbH, B.I.I. *How to use SPIRIVA RESPIMAT*. 2019 2019, March 2019, October 28]; Available from: <https://www.spiriva.com/copd/starting-spiriva/how-to-use-spiriva-respimat>.

4. Australia, N.A.C. *How to use Turbuhaler*. 2017 2017, September 2019, October 28]; Available from: <https://www.nationalasthma.org.au/living-with-asthma/how-to-videos/using-your-turbuhaler>.

5. Australia, N.A.C How to use Breezhaler. 2021, July; available from: https://www.nationalasthma.org.au/living-with-asthma/how-to-videos/how-to-use-breezhaler.

**Supplementary Results:**

**Table S7. Inhaled medications administered by the study participants**

| ***Inhaler*** | | ***All participants N = 103*** | ***Control group***  ***N= 52 (50.5%)*** | ***Intervention group***  ***N= 51 (49.5%)*** |
| --- | --- | --- | --- | --- |
| **pMDI** | Ventolin and Foster | 52 (50.5%) | 29 (55.8%) | 23 (45.1%) |
| **Accuhaler** | Seretide | 40 (38.8%) | 23 (44.2%) | 17 (33.3%) |
| **Respimat** | Spiriva Respimat | 29 (28.2%) | 14 (26.9%) | 15 (29.4%) |
| **Turbohaler** | Symbicort | 58 (56.3%) | 23 (44.2%) | 35 (68.6%) |
| **Breezhaler** | Onbreez,Seebri  And Ultibro | 4 (3.88%) | 3 (5.76%) | 1 (1.96%) |
| **Nebulizers** | Combivent | 12 (11.7%) | 7 (13.5%) | 5 (9.8%) |
|  | Pulmicort | 13 (12.6%) | 9 (17.3%) | 4 (7.8%) |

All data expressed as N (%)

**Table S8. Comorbidities of the study participants**

| ***Characteristic^a^*** | ***All participants N = 103*** | ***Control group***  ***N= 52 (50.5%)*** | ***Intervention group***  ***N= 51 (49.5%)*** | ***P-value*** |
| --- | --- | --- | --- | --- |
| **GERD**  Yes  No | 45 (43.7%)  58 (56.3%) | 21 (46.2%)  31 (53.8%) | 24 (47%)  27 (53%) | 0.495^c^ |
| **Rhinitis**  Yes  No | 61 (59.2%)  42 (40.8%) | 29 (55.8%)  23 (44.2%) | 32 (62.7%)  19 (37.3%) | 0.471^c^ |
| **OSA**  Yes  No | 8 (7.8%)  95 (92.2%) | 3 (5.8%)  49 (94.2%) | 5 (9.8%)  46 (90.2%) | 0.444^c^ |
| **Anxiety**  Yes  No | 4 (3.9%)  99 (96.1%) | 1 (2%)  51 (98%) | 3 (6%)  48 (94%) | 0.298^c^ |
| **Depression**  Yes  No | 3 (2.9%)  100 (97.1%) | 1 (2%)  51 (98%) | 2 (3.9%)  49 (96.1%) | 0.546^c^ |
| **CVD**  Yes  No | 8 (7.8%)  95 (92.2%) | 4 (7.7%)  48 (92.3%) | 4 (7.8%)  47 (92.2%) | 0.977^c^ |
| **Osteoporosis**  Yes  No | 1 (0.97%)  102 (99.03%) | 1 (2%)  51 (98%) | 0 (0%)  51 (100%) | 0.32^c^ |
| **Bronchiectasis**  Yes  No | 3 (2.9%)  100 (97.1%) | 1 (1.92%)  51 (98.8%) | 2 (3.92%)  49 (96.08%) | 0.546^c^ |
| **Action plan**  Yes  No | 58 (56.3%)  45 (43.7%) | 27 (52%)  25 (48%) | 31 (60.7%)  20 (39.3%) | 0.365^c^ |

^a^ All data expressed as N (%) unless otherwise indicated

^c^Chi square test
